# Dietary cholesterol does not break your heart but kills your liver

**DOI:** 10.1016/j.pbj.0000000000000012

**Published:** 2019-06-29

**Authors:** Gerhard P. Püschel, Janin Henkel

**Affiliations:** Department of Nutritional Biochemistry, University of Potsdam, Institute of Nutritional Science, Nuthetal, Germany.

**Keywords:** non-alcoholic fatty liver disease, non-alcoholic steatohepatitis, non-alcoholic fatty liver disease, non-alcoholic steatohepatitis, poly-unsaturated fatty acids, Western-type diet

## Abstract

It is increasingly accepted that dietary cholesterol has a much lower impact on the progression of cardiovascular disease than previously assumed. However, both animal experiments and human studies seem to support the view that dietary cholesterol may contribute to the transition from benign steatosis to the potentially fatal non-alcoholic steatohepatitis. Cholesterol esters and cholesterol accumulate in the hepatocyte and impair its function. This leads to oxidative stress and endoplasmic reticulum stress triggering the release of pro-inflammatory cytokines and rendering the hepatocyte more susceptible to apoptotic or necrotic cell death. Kupffer cells group around dying hepatocytes and phagocytose the hepatocyte debris and lipids. In addition, they are exposed to lipid peroxidation products released from hepatocytes. Kupffer cells, thus activated, release pro-inflammatory, chemotactic and profibrotic cytokines that promote inflammation and fibrosis. Therefore, dietary cholesterol may be harmful to the liver, in particular when administered in combination with polyunsaturated fatty acids that favor lipid peroxidation.

## Atherosclerosis and dietary cholesterol: a historical overview

At the beginning of the last century the impact of dietary lipids on the development of cardiovascular diseases was recognized.^1^ In the 1950s, the comparison of the diet composition at the beginning of the century with post World-War II diets revealed that, among others, increased consumption of saturated fat and cholesterol coincided with the increasing prevalence of cardiovascular disease.^2^ While it was emphasized early on that the ingestion of saturated fatty acids in particular might drive the elevation of plasma cholesterol levels, a reduction of cholesterol consumption was regarded as an effective intervention to reduce plasma cholesterol levels and hence the risk for cardiovascular disease.^3^ This view was supported by a large number of animal experimental models (references in^4–6^), in which high cholesterol diets were used to induce atherosclerotic alterations. In some studies, atherosclerotic lesions could be partially reverted by subsequently feeding a cholesterol-free diet, for example.^7^ In humans, large epidemiological studies revealed high plasma cholesterol, in particular LDL cholesterol, as a major risk factor for the development of atherosclerosis and it was shown that an increase in cholesterol consumption resulted in a proportional increase in plasma cholesterol.^8^ However, the dependency of plasma cholesterol was particularly prominent at very low dietary cholesterol intake, far below the quantities normally found in a typical diet in industrialized countries. In addition, although dietary cholesterol intake resulted in an increase in plasma cholesterol levels, the relative changes were in the range of merely 10%. These considerations shed some doubt on the validity of the recommendation to reduce plasma cholesterol levels by dietary interventions.^9^

## Current view on dietary cholesterol and cardiovascular disease

Critical reevaluation of older data together with new studies that were corrected for potential confounders, which were not considered in the early epidemiological studies, refuted the hypothesis that dietary cholesterol has a major impact on the development of cardiovascular disease,^10^ although this view is not un-contradicted.^11^ Rather than dietary cholesterol itself, other nutritional factors that coincide with the uptake of dietary cholesterol in a diet rich in animal protein appear to be of relevance.^12^ Therefore, current dietary recommendations include a reduction of the intake of animal products and an increase in the intake of whole grains. Notably, the replacement of saturated fatty acids by mono- and polyunsaturated fatty acids in the diet is part of the current recommendations (eg, see healthy eating at http://www.heart.org).^13–15^

## Physiological role of liver in cholesterol metabolism

**Figure 1:**
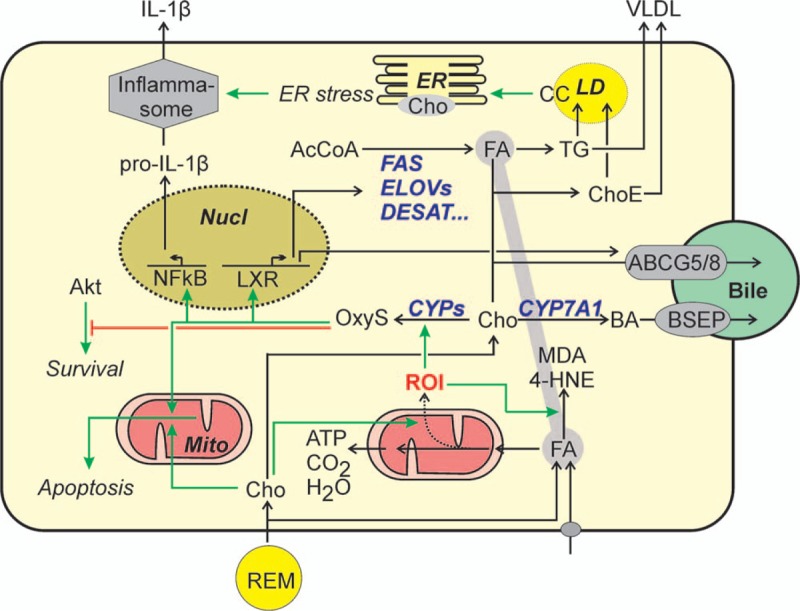
Cholesterol-dependent inflammatory response and cell death in the hepatocyte. Cholesterol and fatty acids are delivered to the hepatocyte from remnant particles or as free fatty acids after increased lipolysis in the adipose tissue. The increased flux of fatty acids through mitochondrial oxidation alongside cholesterol-induced mitochondrial dysfunction result in an increased burden of reactive oxygen intermediates. In particular in the presence of ω-6-polyunsaturated fatty acids, this leads to an increased production of lipid peroxidation products such as malondialdehyde or 4-hydroxynonenal. Cholesterol may be excreted into the bile via ABCG5/8 or after conversion into bile acids via the bile salt export pump. Alternatively, it may react with fatty acids to inert cholesterol esters that are either exported together with triglycerides into VLDL or, if VLDL production reaches its limit, stored in lipid droplets. Excess cholesterol is converted into oxysterols, in particular in the presence of reactive oxygen species. Oxysterol may activate the liver X receptor that directly or indirectly triggers the synthesis of ABCG5/8 and enzymes involved in the fatty acid synthesis. The latter contributes further to the lipid burden of the hepatocyte. Oxysterols may also activate NFκB and thereby enhance, for example, the production of pro-IL-1β. At the same time, free cholesterol and cholesterol crystals formed in lipid droplets may cause ER stress and thereby activate the inflammasome, which converts pro-IL-1β into the secreted mature form. Oxysterols and cholesterol may trigger the mitochondrial apoptotic pathway. Oxysterols in addition may inhibit Akt-dependent survival pathways. 4-HNE = 4-hydroxynonenal, ABCG = ATP-cassette transport protein family G, AcCoA = acetyl-coenzyme A, Akt = protein kinase B, BA = bile acids, BSEP = bile salt export pump, CC = cholesterol crystals, Cho = cholesterol, ChoE = cholesterol ester, CYP = cytochrome P 450, DESAT = desaturase, ELOV = elongase, ER = endoplasmic reticulum, FA = fatty acid, FAS = fatty acid synthase, IL = interleukin, LD = lipid droplet, LXR = liver X receptor, MDA = malondialdehyde, Mito = mitochondrium, NFκB = nuclear factor kappa B, Nucl = nucleus, OxyS = oxysterole, REM = remnant particle (chylomicron remnant, intermediary density lipoprotein), ROI = reactive oxygen intermediates, TG = triglyceride, VLDL = very low density lipoprotein.

The liver plays a central role in cholesterol metabolism. Dietary cholesterol is delivered to the circulation via the chylomicron pathway. The majority of the triglycerides of the chylomicrons are hydrolyzed by lipoprotein lipase that releases fatty acids for their use primarily in adipose tissue and skeletal muscle. The remaining remnant particles, the chylomicron remnants, are rich in cholesterol. Most of these remnant particles are taken up by hepatocytes by receptor-mediated endocytosis among other routes via ApoE and the LDL receptor related protein (Fig. 1). After lysosomal degradation, cholesterol is funneled into different pathways in the hepatocyte. Besides degradation and elimination (see below), cholesterol and cholesterol esters are incorporated into VLDL particles, which are secreted by the hepatocyte. In the periphery, lipoprotein lipase hydrolyzes most of the triglycerides in VLDL as described for chylomicrons and another remnant particle, the IDL, is generated. IDL travels to the liver and is subject to 2 completely different fates: (1) it can be taken up by receptor mediated endocytosis via the LDL receptor or the LDL receptor-related protein as described for the chylomicron remnant or (2) hepatic lipase hydrolyzes a large part of the triglycerides remaining in the IDL particle. While the fatty acids thus liberated are either re-incorporated in triglycerides of VLDL or oxidized by the hepatocyte, the extracellular remains of the IDL are converted into cholesterol-rich LDL particles, which, after leaving the liver, may serve as a source for cholesterol in any cell of the body. If the supply of cholesterol in cells exceeds their demand, they may rid themselves of excess cholesterol by transferring it on HDL. HDL in turn is delivered to the hepatocyte, which can either take up the entire HDL particle by receptor-mediated endocytosis for example via the LDL receptor or extract cholesterol from the cholesterol esters contained in the HDL particle.

Next to the intestinal epithelial cells, the hepatocyte is probably the only site at which significant quantities of cholesterol may be removed from the body either by excretion in form of free cholesterol or by secretion after conversion into bile acids. If the supply with cholesterol exceeds the hepatocyte's capacity for bile acid synthesis and cholesterol secretion, the only safe mode of disposing cholesterol is the formation of cholesterol esters that are transiently stored in the hepatocyte.

## Evidence for the impact of dietary cholesterol on NASH development

Non-alcoholic fatty liver disease (NAFLD) is the hepatic manifestation of the metabolic syndrome.^16^ Its prevalence is increasing as a result of the increasing proportion of overweight and obese patients in the population. While simple steatosis, albeit of clinical significance, appears to be fully reversible, more severe forms of the disease, the non-alcoholic steatohepatitis (NASH), is a chronically progressive disease leading to fibrosis, cirrhosis, and eventually hepatocellular carcinoma. Currently, NASH is the most common reason for terminal hepatic failure in western societies.^17^ Despite intense research, it is not clear (1) whether NAFLD and NASH are different temporal stages of the same disease and if so (2) what are the molecular mechanisms that trigger the progression. Recent evidence suggests that dietary cholesterol might play a critical role in this process.

The impact of dietary cholesterol on liver pathology was actually described prior to its role in the development of atherosclerosis.^1^ In his seminal work on atherosclerosis Anitschkow describes previous work in which the feeding of egg yolk to rabbits resulted in “an extraordinarily rich infiltration of the liver parenchyma with fat-like substances” that was always accompanied by “strongly pronounced areas of parenchymal degeneration”. However, this aspect of dietary cholesterol largely fell into oblivion. Only with the recent surge of NASH and the search for an appropriate rodent model of NASH, renewed interest in the impact of dietary cholesterol on hepatic steatosis and inflammation awoke. While many animal models that are based on diets which induce conditions resembling the metabolic syndrome also result in hepatic steatosis in rodents, most of these diets fail to cause hepatic inflammation and fibrosis in animals. On the other hand, dietary interventions that reproducibly induce hepatic inflammation and fibrosis, such as a choline-methionine-deficient diet, fail to reproduce the symptoms of the metabolic syndrome, indicating that the mechanisms that trigger fibrosis development differ from those in human NASH.^18^

Recently, fructose and cholesterol have been shown to be crucial components in so called Western-type diets for the induction of NASH-like hepatic pathologies in rodents.^19–22^ Feeding a “fast food” diet to mice, which is rich in saturated fat and cholesterol and fructose, resulted in a steady accumulation of cholesterol in the liver over a period of 36 weeks that was accompanied by inflammation and fibrosis.^23^ Notably, insulin resistance preceded hepatic inflammation in these animals. The combination of butter fat with cholesterol in the diet resulted in a NASH-like phenotype in mice with an atherosclerosis-prone genetic background.^24^ While feeding a high fat diet consisting mostly of saturated and mono-unsaturated fatty acids resulted in steatosis, only the combination of the same high fat diet with cholesterol caused inflammation and fibrosis and a highly pronounced hepatic lipid accumulation in male C57BL/6 mice.^25^ Similarly, feeding a Western-type diet containing soybean oil with high amounts of ω-6-PUFA and 0.75% cholesterol induced insulin resistance and a NASH-like hepatic phenotype in mice receiving this diet for 20 weeks. Dietary cholesterol was essential for the development of the NASH phenotype because a soybean oil-containing Western-type diet without cholesterol induced only mild steatosis but failed to induce hepatic inflammation and fibrosis.^26^ Dietary cholesterol caused NASH with a threshold of 0.5%. The maximal inflammatory response determined as hepatic expression of genes for the chemotactic protein MCP1 or the macrophage marker F4/80 was observed at 0.75%; at the highest cholesterol dose of 1% the observed response was weaker.^27^ Along the same lines, gut-specific transgenic expression of SREBP2 further increased the delivery of cholesterol to the liver on a high fat/high cholesterol diet and thereby aggravated and accelerated the development of inflammation and fibrosis compared to wild type animals.^28^

Dietary cholesterol as a component of high fat diets has been shown to trigger NASH development in other animal species as well. Liver damage was also observed in guinea pigs^29,30^ fed a high fat diet rich in cholesterol. In rats^31^ advanced stages of NASH with severe fibrosis were only induced by high fat diets containing cholesterol but not by low cholesterol diets of otherwise identical composition. Feeding a diet with a high content of saturated fat, cholesterol and fructose resulted in NASH in a porcine model.^32^ In a different study using the same porcine model, it was shown that while an atherogenic diet high in fructose, cholesterol and cholate induced hepatic steatosis, a reduction of choline from 900 to 700 ppm with an accompanying increase in methionine content from 2100 to 3900 ppm in the diet (modified atherogenic diet) was necessary to induce inflammation and fibrosis on top of steatosis.^33^ A diet rich in fructose alone caused neither steatosis nor inflammation in this animal model. In a rabbit model the necessity of dietary cholesterol for the induction of a NASH-like liver pathology by a high fat diet was demonstrated by attenuating the diet-induced steatosis, inflammation and fibrosis by simultaneous administration of the cholesterol uptake inhibitor ezetimibe.^34^

In support of the physiological relevance of these findings also in humans, treatment of dylipidemic patients with a combination of statins, which inhibit endogenous cholesterol synthesis, and ezetimibe, a cholesterol uptake inhibitor, to reduce plasma LDL cholesterol levels, improved hepatic steatosis and balooning as well as plasma levels of AST, ALT and γGT in several studies.^35^ However, currently it is not clear whether these effects can be solely attributed to the inhibition of enteral cholesterol uptake by ezetimibe.^36^ In further support of the relevance of dietary cholesterol for the development of NASH, it has recently been shown that the consumption of eggs was positively associated with NAFLD risk.^37^ Thus, while it is increasingly recognized that, at odds with previous assumptions, dietary cholesterol is only of minor relevance for the development of cardiovascular disease,^38–40^ dietary cholesterol appears to be a relevant player in the development of NASH. However, the underlying mechanisms are not yet clear.

## Potential molecular mechanisms underlying NASH-induction by cholesterol

### Cholesterol accumulation in the hepatocyte

Dietary cholesterol accumulates preferentially in the liver.^27^ Although the hepatic accumulation appears to be independent of food composition and can be observed in animals fed cholesterol on a chow-based diet, the accumulation is particularly pronounced in animals receiving a soybean-based diet rich in polyunsaturated fatty acids^41^ (Table 1). Initially, the primary site of cholesterol accumulation is the hepatocyte. In a healthy hepatocyte the endogenous production of cholesterol is reduced when the exogenous supply increases by retaining the inactive form of SREBP2, the key transcription factor controlling the expression of enzymes involved in cholesterol synthesis, in the endoplasmic reticulum (ER). This feedback regulation may be impaired in patients with NASH.^42^ The hepatocyte can handle cholesterol in 3 ways (Fig. 1): (1) cholesterol can be excreted into the bile by active transport via the ABCG5/ABCG8 heterodimer in the apical membrane.^43^ (2) Alternatively, cholesterol can be oxidized by CYP7A1 and/or CYP27A1 to initiate bile acid synthesis.^44^ Synthesis of bile acids and biliary secretion of bile acids and cholesterol is the major route by which the body can dispose of cholesterol. Of note, a large proportion of bile acids, and also cholesterol secreted into the bile, reenter the circulation after reabsorption in the gut. The main impact of inhibitors of the intestinal cholesterol uptake, like ezetimibe, is the interruption of cholesterol re-uptake.^45^ (3) Thirdly, cholesterol can be converted into cholesterol esters which either are incorporated into VLDL together with free cholesterol or may be stored transiently in lipid droplets of the hepatocyte. The latter fate is the only safe way the hepatocyte can dispose of excess cholesterol when the supply exceeds the capacity of turnover in the other routes. Accordingly, the hepatocyte seems to redirect fatty acids from triglyceride synthesis to the synthesis of cholesterol esters, which contributes to the drop of plasma triglycerides after cholesterol feeding observed in many studies^18,26,27,46^ (Table 1) as well as the increase in cholesterol in the VLDL and remnant fractions.^46^ Accumulation of cholesterol ester is further favored because the hepatocyte may react with an increase in de novo fatty acid synthesis in order to ensure a sufficient supply of fatty acids for cholesterol esterification. To this end, cholesterol, after enzymatic or non-enzymatic conversion into oxysterols, may induce SREBP1c and thus enzymes of fatty acid synthesis by activation of the liver X receptor (LXR) (Fig. 1).^47^ However, the accumulation of cholesterol esters per se is unlikely to account for the transition from steatosis to NASH and fibrosis. Rather, oxidative stress resulting from the excessive lipid accumulation or the accumulation of free cholesterol might be relevant in this respect.

**Table 1:**
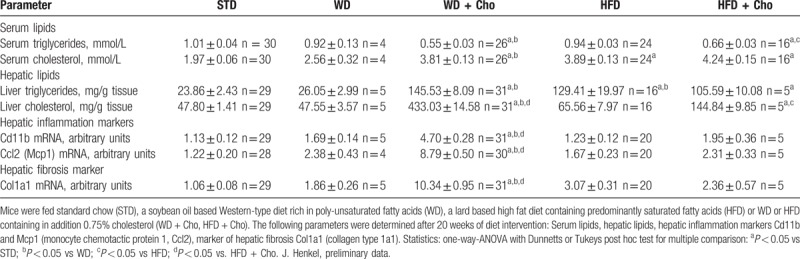
Impact of diets on serum lipids, hepatic lipid accumulation, hepatic inflammation and hepatic fibrosis.

### Oxidative stress

Oxidative stress has been proposed as a possible contributor to the transition from benign steatosis to NASH with inflammation and fibrosis.^48^ Fatty acid oxidation products are elevated in the circulation of patients with NASH in comparison to patients with blunt steatosis^49^ and lipid peroxidation products like malondialdehyde or 4-hydroxynonenal are capable of triggering inflammation and fibrosis^50,51^ by directly activating non-parenchymal cells (see below). Oxidative stress may result from an imbalance between the antioxidative defense systems and the increasing production of reactive oxygen species and lipid peroxidation products in mitochondrial, peroxisomal and microsomal fatty acid oxidation that result from lipid accumulation in hepatic steatosis (Fig. 1).^52^ Cholesterol contributes to the increase in oxidative stress. In particular when administered in combination with ω-6 PUFA, dietary cholesterol appears not only to be a strong trigger of hepatic steatosis, but also for oxidative stress and subsequent inflammation and fibrosis in rodent liver.^26^ Malondialdehyde, which is produced during peroxidation of PUFA under relatively mild oxidative conditions, was increased in livers of mice fed a diet rich in ω-6 PUFA, irrespective of the presence of cholesterol. By contrast, the additional presence of cholesterol caused a strong increase in oxidized peroxiredoxins and protein carbonyls, which are indicative of severe oxidative stress. Notably, it was the combination of PUFA and cholesterol that apparently was responsible for the strong oxidative stress because the same quantity of cholesterol in combination with saturated fat caused steatosis, but only mild signs of inflammation, and no signs of fibrosis (Table 1).

Cholesterol itself is also subject to oxidative modifications. Oxysterols are elevated in NAFLD patients^53^ and appear to be causative in NASH development.^44^ While oxysterols via the LXR induce pathways that eliminate cholesterol from the cell and thereby reduce the cell's cholesterol burden (Fig. 1), depending on the species oxysterols also may have adverse effects. Thus, 25-hydroxy-cholesterol has been shown to enhance the inflammatory response in hepatocytes by NFκB activation^54^ whereas its conjugation product, 25-hydroxycholesterol-3-sulfate attenuated inflammation. Several oxysterols can induce apoptosis by triggering the mitochondrial apoptotic pathway^55^ in hepatoma cells or primary rat hepatocytes if cells were exposed to a combination of oxysterols and fatty acids. In addition, oxysterols appear to contribute to cell death by antagonizing Akt-dependent survival pathways (Fig. 1).^56^ Although in a different study, oxysterols apparently did not reduce cell viability of hepatocytes,^57^ they still might contribute to NASH development by acting on non-parenchymal liver cells (see below).

### Free cholesterol as trigger of hepatocyte apoptosis and necrosis

Cholesterol may be safely stored in cholesterol esters. However, this storage is impaired in NASH patients. In addition to the impaired feedback inhibition of cholesterol synthesis (see above), an increase in the activity of cholesterol ester hydrolase may contribute to the increase in free cholesterol.^58^ The concentration of free cholesterol increases as liver damage advances.^59^

Changes in free cholesterol may result in ER stress.^42,60^ ER stress induced activation of the IRE1α-XBP-1 pathway can further promote steatosis by inducing key enzymes of triglyceride biosynthesis. In addition, ER stress may result in the activation of the inflammasome (Fig. 1) and a subsequent increase in IL-1β production in hepatocytes,^61^ directly linking cholesterol accumulation to the induction of an inflammatory response. Furthermore, cholesterol-elicited ER stress may trigger hepatocyte apoptosis or sensitize hepatocytes to other proapoptotic signals.^42^ In a different study, no ER stress-mediated activation of apoptotic pathways was observed. Rather, accumulation of free cholesterol in mitochondria caused a depletion of mitochondrial reduced glutathione and sensitized hepatocytes against TNFα or FAS-induced apoptosis and necrosis thereby fostering NASH development.^62^

**Figure 2:**
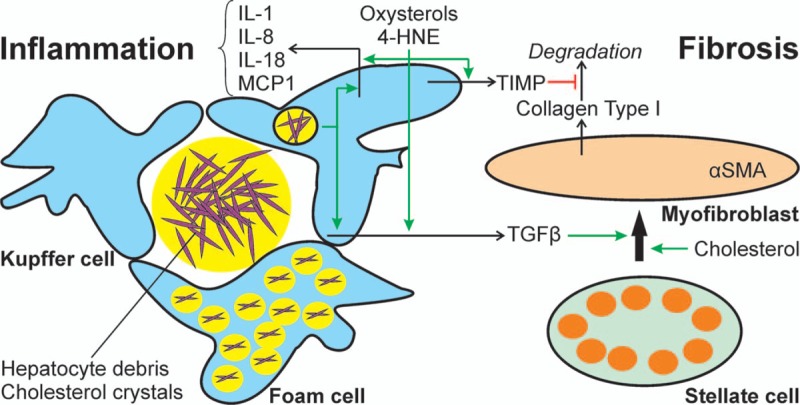
Cholesterol-dependent activation of non-parenchymal liver cells. Kupffer cells form crown-like structures around hepatocyte debris and lipid droplets. Lysosomal enzymes cleave cholesterol esters, allowing the formation of cholesterol crystals. Kupffer cells phagocytose hepatocyte debris, lipids and cholesterol crystals and thereby are activated to produce pro-inflammatory and pro-fibrotic cytokines. Ultimately, Kupffer cells turn into lipid-laden foam cells. Oxysterols and lipid-peroxidation products released from hepatocytes may further stimulate the release of pro-inflammatory and pro-fibrotic cytokines from Kupffer cells, as well as the production of tissue inhibitors of metalloproteinases that inhibit the degradation of extracellular matrix proteins. Cholesterol and Kupffer cell-derived TGFβ favor the transdifferentiation of stellate cells to myofibroblasts, which produce excessive amounts of extracellular matrix proteins, promoting the development of fibrosis. 4-HNE = 4-hydroxynonenal, IL = interleukin, MCP1 = monocyte chemoattractant protein 1, TGFβ = transforming growth factor β, TIMP = tissue inhibitor of metalloproteinases.

Recent evidence suggests that an increase in intracellular free cholesterol may affect the regulation of lipid turnover by interfering with the function of proteins in the lipid droplet coat. Formation of cholesterol crystals within the phospholipid monolayer surrounding the lipid droplet was observed^63^ and correlated with the progression of steatosis to NASH. While the initial formation of cholesterol crystals within the hepatocyte appeared to promote hepatocyte death, the remnant lipid droplets of dead hepatocytes were surrounded by Kupffer cells in crown-like structures. While cholesterol crystals were found only in the outer layer of lipid droplets within the hepatocytes, presumably due to further hydrolysis of cholesterol esters by Kupffer cell lysosmal enzymes lipid droplet remnants within the crown-like structures contained cholesterol crystals not only in the lipid droplet coat but also in their core.^27^ The Kupffer cells phagocytosing the cholesterol crystals evolve into foam cells and react with an inflammatory response (Fig. 2).

### Kupffer cell and stellate cell activation

Cholesterol crystals may trigger the inflammatory response in THP macrophages^27^ or primary Kupffer cells,^26^ phagocytosing lipid droplets of apoptotic or necrotic hepatocytes. Lipolytic enzymes released in the zone of inflammation may release cholesterol from cholesterol esters and thereby enhance cholesterol crystal formation (Fig. 2). Transwell experiments showed that direct contact and phagocytosis of the crystals was mandatory. Cholesterol crystals can activate the NLRP3 inflammasome and thereby promote the activation of IL-1β and IL-18 from their precursors. Consequently, inhibition of the NLRP3 inflammasome reduced the severity of liver inflammation and fibrosis in genetic or diet-induced mouse models of NASH.^64^ Cholesterol has been shown to favor the transdifferentiation of hepatic stellate cells into myofibroblasts (Fig. 2) and thereby might contribute to the development of hepatic fibrosis.^65^

As noted above, oxidative stress is a crucial factor in the development of NASH. Apart from direct damage to the hepatocyte, lipid oxidation products may activate the inflammatory response in Kupffer cells. Thus, 27-hydroxycholesterol in combination with 4-hydroxynonenal, both of which are products of lipid oxidation, may activate TLR4 signaling and cause NFκB activation in animal models of atherosclerosis. A similar mechanism has been proposed as potential mechanism contributing to the inflammation in NASH (Fig. 2).^48^ In addition, oxysterols increased TGFβ and MCP1 expression in Kupffer cells as well as IL-8 and TIMP secretion from hepatic stellate cells and thereby may contribute to inflammation and fibrosis.^57^

## Concluding remark

While dietary cholesterol apparently has a much lower impact on the progression of cardiovascular disease than previously assumed, both animal experiments and human studies seem to support the view that dietary cholesterol may contribute to the transition from benign steatosis to the potentially fatal NASH. Dietary cholesterol may be harmful to the liver, in particular when administered in combination with polyunsaturated fatty acids, which favor lipid peroxidation.^26^ This finding is of particular relevance, considering recent recommendations to replace saturated fat with polyunsaturated fat for the prevention of cardiovascular disease without explicitly suggesting a concurrent reduction of cholesterol intake.^66^

## Acknowledgments

The literature list in this short review is far from comprehensive. Many authors’ relevant work was not cited. Please accept our sincere apologies.

## Funding

The authors’ research was funded in part by the DFG grant HE 7032/1-1.

## Conflicts of interest

The authors have no conflict of interests to declare.
